# Can Biomarker-Based Monitoring Detect the Early Development of Diastolic Dysfunction During Chemotherapy?

**DOI:** 10.3390/medicina62091634

**Published:** 2026-08-26

**Authors:** Anca Daniela Farcaş, Cerasela Mihaela Goidescu, Mirela Anca Stoia, Florin Petru Anton, Andrada Viorica Pârvu, Camil Horia Eusebiu Crişan, Diana Larisa Mocan Hognogi

**Affiliations:** 1 Internal Medicine Department, “Iuliu Haţieganu” University of Medicine and Pharmacy, 400012 Cluj Napoca, Romania; ancafarcas@yahoo.com (A.D.F.); mirelastoia@yahoo.com (M.A.S.);; 2 Cardiology Department, Cluj County Emergency Clinical Hospital, 400347 Cluj Napoca, Romania; 3 “Constantin Papilian” Military Emergency Clinical Hospital, 400132 Cluj Napoca, Romania; 4 Oncological Department, “Iuliu Haţieganu” University of Medicine and Pharmacy, 400012 Cluj Napoca, Romania; parvuandrada@hotmail.com; 5 “Prof. Dr. Ion Chiricuță” Oncology Institute Cluj, 400015 Cluj Napoca, Romania

**Keywords:** diastolic disfunction, chemotherapy, heart failure

## Abstract

*Background and Objectives:* Although modern oncologic therapies have substantially improved cancer survival and overall prognosis, they have also led to an increasing burden of cardiovascular toxicity. Preventing severe complications such as heart failure and death remains a major priority, while maintaining the need for effective and potentially curative cancer therapy. Careful patient monitoring and early detection of cardiovascular complications may allow the timely implementation of cardioprotective strategies and treatments that could delay or prevent myocardial toxicity. *Materials and Methods:* A total of 92 women with breast cancer were enrolled in a prospective observational cohort study. All patients received an anthracycline-based chemotherapy regimen, cyclophosphamide, docetaxel and trastuzumab. Biomarker assessment, including NT-proBNP, high-sensitivity cardiac troponin I (hs-cTnI), Gal-3, and GDF-15, was performed at baseline and at the initiation of trastuzumab-based therapy. A comprehensive diastolic function assessment was performed, including transmitral Doppler flow parameters and tissue velocities. Patients were followed for 12 months, and all cardiovascular events occurring during the follow-up period were recorded. *Results:* Biological and ecocardiographic parameters were analyzed and multiple models of prediction were made. To identify predictors of estimated left ventricular filling pressure (eLVFP), stepwise multiple linear regression analyses were performed, and four significant predictors of left ventricular end-diastolic filling pressure were identified: one echocardiographic parameter (baseline LV filling pressure) and three biological variables (changes in Gal-3, GDF-15, and hs-cTnI levels after treatment). The overall regression model was highly significant (*p* < 0.001) and explained 77.07% of the variance in the dependent variable. Gal-3 was the strongest predictor of left ventricular filling pressure (*p* < 0.001). *Conclusions:* The regression analyses suggest that this phenotype is multifactorial, with echocardiographic indices capturing the functional component and circulating biomarkers reflecting complementary aspects of the underlying biological response. This multimodal approach may therefore help identify patients with early treatment-related cardiac changes and potentially those at increased risk of subsequent CTRCD, particularly during a period when conventional LVEF-based surveillance may still appear reassuring.

## 1. Introduction

Cancer therapy-related cardiac dysfunction (CTRCD) is the currently recommended term for the cardiovascular complications associated with anticancer treatment, including cardiac injury, myocardial dysfunction, and heart failure. This spectrum of complications may be induced by a wide range of oncologic therapies, including chemotherapy, immunotherapy, targeted agents, and radiotherapy. The clinical consequences can be life-threatening, disabling, and may limit the continuation of cancer treatment, thereby adversely affecting patient prognosis. Cardiac dysfunction is one of the many long-term complications that cancer survivors may need to cope with after completing treatment.

Although modern oncologic therapies have substantially improved cancer survival and overall prognosis, they have also led to an increasing burden of cardiovascular toxicity. Anthracyclines, together with HER2-targeted therapies and more recent immune checkpoint inhibitors, represent some of the most important causes of cancer therapy-related cardiac dysfunction, often with long-term consequences. Anthracyclines induce left ventricular systolic dysfunction in a dose-dependent and cumulative manner. Clinically overt heart failure occurs in approximately 1–3% of patients receiving standard anthracycline doses, whereas subclinical cardiac dysfunction may be considerably more frequent, particularly among high-risk patients [1,2,3]. HER2-targeted therapies may cause asymptomatic declines in left ventricular ejection fraction in up to 7% of patients treated with trastuzumab alone; however, the incidence may increase to as high as 27% when trastuzumab is administered sequentially or concomitantly with anthracyclines, or when dual HER2 blockade with trastuzumab and pertuzumab is used.

Taxanes, including docetaxel, are generally associated with a lower risk of direct myocardial toxicity than anthracyclines or HER2-targeted therapies. However, cardiovascular adverse events have been described, including arrhythmias, ischemic events, heart failure, and treatment-limiting fluid retention. Older studies reported heart failure rates of up to 2.3–8% in patients receiving taxane-based regimens; however, these estimates were derived from heterogeneous populations and combination chemotherapy protocols, making the specific contribution of taxanes difficult to ascertain [4]. Docetaxel is particularly associated with dose-dependent fluid retention, which may manifest as peripheral edema, pleural or pericardial effusion, weight gain, and, in susceptible patients, heart failure decompensation. The mechanisms of docetaxel-related cardiotoxicity are incompletely defined, but may involve microtubule stabilization within cardiomyocytes, oxidative stress, apoptosis, endothelial dysfunction, and capillary leak. True docetaxel-induced left ventricular systolic dysfunction appears uncommon, and recovery is more likely when the clinical presentation is driven by fluid retention or hypersensitivity rather than irreversible myocardial injury. The risk is higher when docetaxel is administered after or together with anthracyclines or HER2-targeted therapies, and in patients with pre-existing cardiovascular disease [5,6,7].

Preventing severe complications such as heart failure and death remains a major priority, while maintaining the need for effective and potentially curative cancer therapy. Careful patient monitoring and early detection of cardiovascular complications may allow the timely implementation of cardioprotective strategies and treatments that could delay or prevent myocardial toxicity. Identifying readily available biological and imaging markers with predictive value represents an important area of ongoing research that still requires further investigation. This approach may be particularly useful in selected subsets of breast cancer patients, such as pregnant women, for whom certain diagnostic imaging modalities are contraindicated or cannot be safely performed [8].

Although troponin I (hsTnI) and NT-proBNP have shown their usefulness, there is still incomplete data regarding the time and frequency of their testing.

The search for novel and clinically useful biomarkers for the early identification of cardiotoxicity has focused attention on GDF15 (Growth Differentiation Factor 15) and Gal-3 (Galectin-3), two circulating biomarkers widely investigated in inflammatory, cardiovascular, and oncological diseases. GDF15, a stress-response cytokine belonging to the TGF-β superfamily, is associated with metabolic stress, systemic inflammation, and cellular senescence when present at elevated levels. Gal-3, a β-galactoside-binding lectin predominantly secreted by macrophages, plays a central role in fibrosis, inflammation, and cardiac remodeling. It has also been validated as a reliable biomarker for the risk stratification and progression assessment of heart failure [9,10].

Radiotherapy involving the heart, particularly left-sided thoracic irradiation, is an important contributor to cancer therapy-related cardiovascular disease. Unlike anthracyclines, which primarily induce myocardial injury and left ventricular systolic dysfunction, radiotherapy mainly promotes endothelial damage, microvascular dysfunction, accelerated coronary atherosclerosis, pericardial disease, valvular fibrosis, conduction abnormalities, and restrictive or diastolic myocardial dysfunction. The risk is dose-dependent and appears to increase linearly with mean heart dose; in breast cancer survivors, the rate of major coronary events has been reported to increase by approximately 7.4% for each 1 Gy increase in mean heart dose [11]. This risk is particularly relevant after left-sided thoracic exposure and may persist for decades. The cardiovascular burden is further amplified when radiotherapy is combined with cardiotoxic systemic therapies, especially anthracyclines and HER2-targeted agents, as well as by traditional cardiovascular risk factors [12,13].

Diastolic dysfunction may occur early during potentially cardiotoxic chemotherapy, particularly after anthracycline-based regimens. Reported echocardiographic changes include reductions in E/A ratio and tissue Doppler e′ velocities, with variable increases in E/e′ ratio, deceleration time, and isovolumic relaxation time. Although these parameters may precede overt left ventricular systolic dysfunction, their prognostic value is limited by load dependency and heterogeneity across studies. Their clinical relevance appears stronger when combined with myocardial deformation imaging and biomarkers, particularly GLS reduction, NT-proBNP elevation, and troponin release. Therefore, diastolic indices could be considered complementary markers of early cancer therapy-related cardiac dysfunction rather than independent diagnostic or prognostic criteria [14,15,16,17].

The present study aimed to evaluate the utility of circulating biomarkers, including NT-proBNP, high-sensitivity cardiac troponin I (hs-cTnI), galectin-3, and growth differentiation factor 15 (GDF-15), for the early detection of cancer therapy-related cardiac dysfunction in women with breast cancer receiving anthracycline-based chemotherapy followed by docetaxel and trastuzumab. In addition, we investigated the association between changes in these biomarkers and serial echocardiographic parameters, with particular emphasis on early alterations in left ventricular diastolic function, in order to identify potential predictors of subclinical cardiotoxicity.

## 2. Materials and Methods

A total of 92 women with breast cancer were prospectively enrolled in an observational cohort study evaluating early cardiac changes associated with systemic anticancer therapy. All patients initially received an anthracycline-based chemotherapy regimen consisting of epirubicin and cyclophosphamide administered every 3 weeks for four cycles. Subsequently, patients with HER2-positive breast cancer continued treatment with four cycles of docetaxel plus trastuzumab administered at 3-week intervals, whereas patients with HER2-negative disease did not receive anti-HER2 therapy. Accordingly, patients were stratified according to HER2 status and treatment regimen into two groups: those receiving trastuzumab plus docetaxel (HER2-positive breast cancer group) and those not receiving anti-HER2 therapy (HER2-negative breast cancer group).

Consecutive eligible patients meeting the predefined inclusion and exclusion criteria were prospectively enrolled to reduce selection bias and enhance the representativeness of the study population. The sequential exposure to anthracycline-based chemotherapy followed by trastuzumab-based therapy in HER2-positive patients represents a clinically relevant setting for the investigation of early cancer therapy-related cardiac dysfunction.

All participants provided written informed consent and underwent comprehensive clinical, biological, and echocardiographic evaluation. In patients receiving trastuzumab plus docetaxel therapy, biomarker assessment, including NT-proBNP, high-sensitivity cardiac troponin I (hs-cTnI), galectin-3 (Gal-3), and growth differentiation factor 15 (GDF-15), was performed immediately before initiation of trastuzumab plus docetaxel therapy and after completion of the first trastuzumab plus docetaxel cycle, concomitantly with echocardiographic evaluation.

Echocardiographic assessment was performed immediately before initiation of trastuzumab plus docetaxel therapy (baseline) and after completion of the first treatment cycle. Additional follow-up echocardiographic evaluations were performed at 3 and 6 months after completion of oncological treatment.

Venous blood samples were collected after an overnight fast and following 10 min of rest under standardized conditions. Samples for serum biomarker analysis were collected in anticoagulant-free tubes, whereas hs-cTnI measurement was performed from plasma samples collected in anticoagulant-containing tubes. Serum samples were allowed to clot for approximately 30 min and subsequently centrifuged at 1000× *g* for 15 min. Serum and plasma samples were aliquoted and stored at −80 °C until analysis. Repeated freeze–thaw cycles were avoided.

Serum galectin-3 concentrations were measured using a commercially available quantitative sandwich enzyme-linked immunosorbent assay (ELISA) [MyBioSource Human Galectin-3 ELISA Kit—MyBioSource, San Diego, CA, USA], detection range 0.2 ng/mL–60 ng/mL; sensitivity 0.13 ng/mL], according to the manufacturer’s instructions. Samples were diluted as recommended by the manufacturer and analyzed in duplicate. Galectin-3 concentrations were calculated from the standard calibration curve and corrected for the sample dilution factor.

Serum GDF-15 concentrations were determined using a quantitative sandwich ELISA [MyBioSource Human Growth Differentiation Factor 15 ELISA Kit, MyBioSource, San Diego, CA, USA, detection range 31.2 pg/mL–2000 pg/mL; sensitivity < 10 pg/mL], according to the manufacturer’s protocol. Samples, standards, and controls were analyzed in duplicate. Concentrations were calculated using a four-parameter logistic standard curve and multiplied by the relevant dilution factor.

Plasma hs-cTnI levels were measured using a quantitative sandwich ELISA [MyBioSource High Sensitivity Cardiac Troponin I ELISA Kit, MyBioSource, San Diego, CA, USA, detection range 7–1500 ng/L; sensitivity 3.62 ng/L], according to the manufacturer’s protocol. Samples, standards, and controls were analysed in duplicate.

Echocardiographic assessment was performed immediately before initiation of trastuzumab plus docetaxel therapy, after completion of the first trastuzumab plus docetaxel cycle, and at 3 and 6 months after completion of oncological treatment. Echocardiographic examinations were performed using a Philips echocardiography system equipped with a 2–5 MHz transducer by an experienced cardiologist with expertise in echocardiography.

Left ventricular systolic function was assessed by measurement of left ventricular ejection fraction (LVEF) and global longitudinal strain (GLS). A comprehensive assessment of diastolic function was performed using transmitral Doppler parameters and tissue Doppler imaging-derived myocardial velocities obtained from the septal, lateral, anterior, and inferior mitral annulus. Diastolic function parameters included peak early (E) and late (A) transmitral velocities, E/A ratio, deceleration time, isovolumic relaxation time, tissue Doppler-derived e′ velocities, E/e′ ratio, and left atrial volume index, with particular emphasis on estimation of left ventricular filling pressures. Diastolic function assessment was performed according to the 2016 recommendations of the American Society of Echocardiography and the European Association of Cardiovascular Imaging (EACVI) [3].

Statistical analysis was performed using MedCalc Statistical Software, version 23.6.5 (MedCalc Software Ltd., Ostend, Belgium). The distribution of continuous variables was assessed using the Shapiro–Wilk test. Continuous variables are presented as mean ± standard deviation (SD) with 95% confidence intervals (95% CIs) or as median with interquartile range (IQR), according to data distribution. Categorical variables are expressed as frequencies and percentages.

Comparisons between groups were performed using the chi-square test for categorical variables and the Mann–Whitney U test for non-normally distributed continuous and ordinal variables. Linear regression analysis was used to evaluate associations between continuous variables. Multiple linear regression analysis was performed to identify independent predictors of left ventricular filling pressure after the first trastuzumab cycle. Stepwise multiple linear regression analysis was subsequently applied to determine independent predictors of left ventricular filling pressure. Clinically relevant variables and those significantly associated with left ventricular filling pressure in univariate analysis (*p* < 0.05) were considered eligible for inclusion in the multivariable model.

All statistical tests were two-sided, and a *p*-value < 0.05 was considered statistically significant. No missing data were observed; therefore, no imputation methods were required.

## 3. Results

Baseline clinical, demographic, and echocardiographic characteristics are presented in Table 1. No significant differences were observed between patients with HER2-positive and HER2-negative breast cancer regarding demographic characteristics, cardiovascular risk profile, or baseline echocardiographic parameters. Left ventricular systolic and diastolic function indices were within normal limits in both groups at baseline.

Following the first trastuzumab plus docetaxel cycle, numerical changes in NT-proBNP, hs-cTnI, GDF-15, and galectin-3 concentrations were observed; however, none reached statistical significance (Table 2). Similarly, no significant changes were detected in left ventricular systolic function parameters, including left ventricular ejection fraction (LVEF) and global longitudinal strain (GLS). In contrast, a significant increase in estimated left ventricular filling pressure was observed after treatment, reflected by an increase in the E/e′ ratio (*p* < 0.001).

Detailed assessment of diastolic function parameters demonstrated that patients who developed treatment-emergent diastolic abnormalities after the first trastuzumab plus docetaxel cycle exhibited significant alterations in early myocardial relaxation (Table 3). These changes were characterized by a reduction in the E/A ratio, decreased septal and lateral mitral annular e′ velocities, and increased septal and lateral E/e′ ratios, suggesting impaired myocardial relaxation associated with elevated left ventricular filling pressures. Furthermore, the increase in estimated filling pressure was accompanied by significant left atrial remodeling, reflected by an increase in left atrial volume index (LAVi).

**Figure 1:**
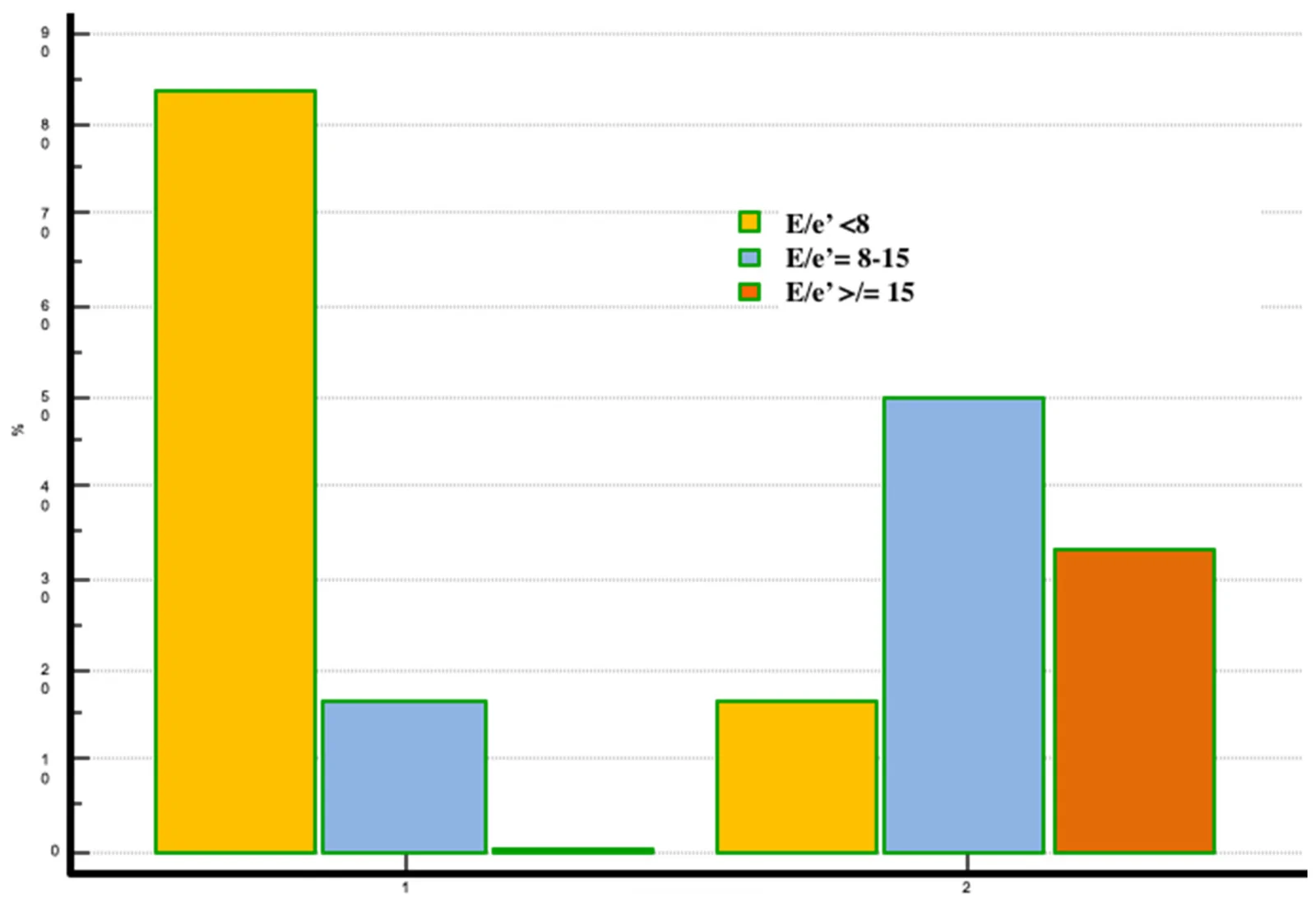
Changes in estimated left ventricular filling pressure based on E/e′ ratio after the first trastuzumab plus docetaxel cycle.

Among patients receiving trastuzumab-based therapy, the distribution of estimated left ventricular filling pressure categories significantly changed after the first treatment cycle (Table 4, Figure 1). At baseline, 83.3% of patients had normal filling pressures (E/e′ < 8), whereas after treatment only 16.7% remained within this category (*p* < 0.0001). Conversely, the proportion of patients with intermediate filling pressures (E/e′ 8–15) increased from 16.7% to 50.0% (*p* < 0.0001). Moreover, 33.3% of patients developed elevated filling pressures (E/e′ ≥ 15) after only one treatment cycle (*p* = 0.017).

To identify independent determinants of early impairment in left ventricular filling pressure, stepwise multiple linear regression analysis was performed in patients receiving trastuzumab-based therapy.

Multiple linear regression analyses were performed in 48 patients receiving trastuzumab-based therapy to identify independent echocardiographic and biochemical predictors of post-treatment E/e′. The characteristics and performance of the four regression models are summarized in Table 5.

The initial echocardiographic model, including DT, RV-RA pressure gradient, and septal e’, was statistically significant and demonstrated a high explanatory capacity, accounting for 76.5% of the variability in post-treatment E/e′ (R^2^ = 0.7652; adjusted R^2^ = 0.7492; *p* < 0.0001). All three echocardiographic variables were independently associated with post-treatment E/e′. Among these variables, septal e’ showed the strongest independent association with post-treatment E/e′ (R partial = 0.8122; *p* < 0.0001).

To assess whether transformation of the dependent variable improved model performance, the same echocardiographic predictors were evaluated using the natural logarithm of post-treatment E/e′. The log-transformed model showed a modest improvement in explanatory capacity, accounting for 78.9% of the variance (R^2^ = 0.7886; adjusted R^2^ = 0.7741; *p* < 0.0001), compared with 76.5% in the untransformed model. Septal e’ continued to demonstrate the strongest partial association with the dependent variable (r partial = −0.8103; *p* < 0.0001).

A separate model was subsequently developed using the biochemical markers hs-cTnI, Gal-3, and GDF-15 as independent variables. This model was also highly significant (*p* < 0.0001) and explained 72.5% of the variability in post-treatment E/e′ (R^2^ = 0.7245; adjusted R^2^ = 0.7058). Hs-cTnI, Gal-3 and GDF-15 showed significant positive associations with post-treatment E/e′. Gal-3 demonstrated the strongest independent association among the three biomarkers (r partial = 0.8101; *p* < 0.0001), indicating that it contributed the largest unique proportion of explained variability within the biomarker-based model.

Finally, a combined model incorporating baseline E/e′ together with hs-cTnI, Gal-3, and GDF-15 was evaluated. This model showed the highest explanatory capacity among the models predicting untransformed post-treatment E/e′, accounting for 79.0% of its variability (R^2^ = 0.7902; adjusted R^2^ = 0.7707; *p* < 0.0001). Baseline E/e′ was positively associated with post-treatment E/e′ (B = 0.2003; 95% CI: 0.09014–0.3104; *p* = 0.0007), indicating that higher baseline filling pressures were independently associated with higher post-treatment filling pressures. Gal-3 remained the biomarker with the strongest partial association in the combined model (r partial = 0.6982; *p* < 0.0001).

Overall, all four regression models were statistically significant and demonstrated substantial explanatory capacity. The biochemical model alone explained 72.5% of the variability in post-treatment E/e′, whereas the addition of baseline E/e′ increased the explained variance to 79.0%. The log-transformed echocardiographic model also showed a slightly greater explanatory capacity than the corresponding untransformed model (adjusted R^2^: 0.7741 vs. 0.7492). These findings indicate that both baseline echocardiographic characteristics and biochemical markers contributed independently to the variability in post-treatment E/e′, with the combined model providing the highest explanatory performance for the untransformed outcome.

## 4. Discussion

The present study investigated whether early changes in diastolic function and circulating biomarkers could identify subclinical cardiac involvement during trastuzumab-based therapy in patients with breast cancer. The main finding was that significant alterations in left ventricular diastolic function occurred after the first treatment cycle despite preserved left ventricular systolic function. In particular, an increase in estimated left ventricular filling pressure, expressed by E/e′, was accompanied by significant reductions in tissue Doppler-derived myocardial relaxation velocities. These changes were more pronounced in patients who developed treatment-emergent diastolic abnormalities. Furthermore, the regression analyses demonstrated that both echocardiographic and biochemical parameters were independently associated with post-treatment E/e′, with the combined model explaining approximately 79% of its variability.

In our cohort, conventional indices of left ventricular systolic function remained preserved after the first treatment cycle, with no significant changes in LVEF or GLS. This finding is clinically relevant because it suggests that the earliest cardiac effects of trastuzumab-based therapy may not necessarily be detected by conventional assessment of systolic function. Although LVEF remains the most widely used parameter for surveillance of cancer therapy-related cardiac dysfunction, its limitations in identifying early myocardial injury are well recognized. GLS may provide greater sensitivity for detecting subclinical systolic dysfunction; however, in the present cohort, GLS also remained stable during the early treatment period. Therefore, the absence of an early decline in LVEF or GLS does not appear to exclude the presence of treatment-related cardiac functional changes.

In contrast, a clear pattern of early diastolic impairment was observed. Estimated left ventricular filling pressure increased significantly after the first treatment cycle, as reflected by the increase in E/e′. This change was accompanied by alterations in several complementary parameters of diastolic function. Among patients who developed treatment-emergent diastolic abnormalities, E/A decreased, septal and lateral e′ velocities decreased, while septal and lateral E/e′ increased. LAVi also increased significantly in this group. The simultaneous changes in transmitral flow, tissue Doppler velocities, estimated filling pressure, and left atrial volume support the interpretation that the observed increase in E/e′ represented a broader alteration in left ventricular diastolic function rather than an isolated change in a single echocardiographic parameter.

The reduction in tissue Doppler-derived myocardial relaxation velocities was particularly consistent. In the trastuzumab-based therapy group, mean e′ decreased significantly after the first treatment cycle, and a similar significant reduction was observed for lateral e′. The majority of patients demonstrated a decrease in mean e′ and lateral e′ after treatment. These findings provide additional evidence that impairment of myocardial relaxation may occur very early during treatment, before detectable changes in conventional systolic function. Importantly, the magnitude and direction of these changes were consistent with the increase in E/e′ observed after treatment, providing physiological support for the interpretation of increased filling pressure.

The subgroup analysis further supports this interpretation. Patients who developed treatment-emergent diastolic abnormalities showed significant reductions in septal and lateral e′ together with increases in septal and lateral E/e′ and LAVi, whereas these changes did not reach statistical significance among patients without treatment-emergent diastolic abnormalities. Thus, the development of diastolic abnormalities was characterized by a coherent pattern of impaired myocardial relaxation and increased estimated filling pressure. These findings suggest that diastolic dysfunction may represent an early manifestation of cardiac involvement during trastuzumab-based therapy, even when systolic function remains preserved.

The early increase in estimated left ventricular filling pressure observed in our study is of particular interest. Approximately one-third of patients developed elevated filling pressures after the first trastuzumab plus docetaxel cycle. This finding suggests that changes in ventricular relaxation and compliance may occur relatively early during exposure to anti-HER2 therapy. Although E/e′ should not be interpreted in isolation, the concomitant reduction in e′ and increase in LAVi strengthen the interpretation of these findings as an early alteration in diastolic function. Therefore, assessment of diastolic parameters may provide additional information that is not captured by conventional systolic surveillance alone.

These findings are consistent with the concept that cancer therapy-related cardiac dysfunction represents a continuum in which abnormalities in myocardial relaxation, ventricular filling, and myocardial deformation may precede overt reductions in LVEF. Previous studies, including SAFE-HEaRt, SUCCOUR, and PRADA, have emphasized the importance of identifying subtle cardiac changes before clinically significant systolic dysfunction becomes apparent. Our findings extend these observations by suggesting that alterations in diastolic function may also occur at a very early stage of trastuzumab-based treatment. Rather than considering preserved LVEF and GLS as evidence of complete cardiac stability, the present findings support a more comprehensive echocardiographic assessment that also considers diastolic function.

The regression analyses provide further support for the relevance of these early echocardiographic changes. The initial echocardiographic model explained 76.5% of the variability in post-treatment E/e′, with all three included echocardiographic variables remaining independently associated with the outcome. Septal e′ demonstrated the strongest independent association with post-treatment E/e′. The negative regression coefficient indicates that lower post-treatment septal e′ was associated with higher post-treatment E/e′. This finding is consistent with the paired analysis showing a significant reduction in tissue Doppler-derived e′ after treatment and supports the physiological relationship between impaired myocardial relaxation and increased estimated filling pressure.

The importance of septal e′ was maintained when the dependent variable was log-transformed. The log-transformed echocardiographic model showed a modest improvement in explanatory capacity, with R^2^ increasing from 0.7652 to 0.7886 and adjusted R^2^ from 0.7492 to 0.7741. Septal e′ remained the strongest independent echocardiographic correlate. The persistence of this relationship after transformation supports the robustness of the association and suggests that the observed relationship was not substantially dependent on the distribution of the post-treatment E/e′ variable.

The biochemical model provided complementary information. hs-cTnI, Gal-3, and GDF-15 together explained 72.5% of the variability in post-treatment E/e′. Among these biomarkers, Gal-3 demonstrated the strongest independent association. This finding is of particular interest because Gal-3 has been associated with myocardial remodeling, fibrosis, inflammation, and extracellular matrix turnover. Its strong association with post-treatment E/e′ may therefore reflect biological processes related to changes in myocardial compliance and ventricular filling that are not directly captured by conventional echocardiographic measurements.

GDF-15 also remained independently associated with post-treatment E/e′. Although the overall changes in circulating biomarkers after the first treatment cycle did not reach statistical significance, the regression analysis demonstrated that individual differences in biomarker levels were associated with subsequent variation in estimated filling pressure. This distinction is important. A biomarker may fail to demonstrate a significant change at the group level while still providing clinically relevant information regarding interindividual susceptibility to treatment-related cardiac changes. The regression findings therefore suggest that the predictive value of these biomarkers may be more evident when individual variability is taken into account rather than through simple pre-treatment versus post-treatment comparisons.

The combined model, incorporating baseline E/e′ together with hs-cTnI, Gal-3, and GDF-15, demonstrated the highest explanatory capacity among the models predicting untransformed post-treatment E/e′, accounting for 79.0% of its variability. Baseline E/e′ was independently associated with post-treatment E/e′, indicating that patients with higher baseline filling pressures tended to remain at greater risk of increased filling pressures after treatment. Gal-3 remained the biomarker with the strongest partial association in the combined model. These findings suggest that baseline echocardiographic characteristics and treatment-related biochemical changes provide complementary information regarding the individual cardiac response to trastuzumab-based therapy.

The difference between the echocardiographic and biochemical models is also relevant. The echocardiographic model showed a slightly greater explanatory capacity than the biomarker-only model, whereas addition of baseline E/e′ to the biomarker variables increased the explained variance to 79.0%. This suggests that the assessment of early treatment-related changes in filling pressure may benefit from combining functional imaging parameters with biomarkers reflecting myocardial injury and remodeling. Importantly, the results do not suggest that biomarkers should replace echocardiographic assessment. Rather, they support their potential complementary role in identifying patients with early cardiac changes that may not yet be accompanied by a decline in LVEF or GLS.

The findings also provide a possible explanation for the discrepancy between preserved systolic function and early diastolic abnormalities observed in our cohort. The absence of significant changes in LVEF and GLS, together with the reduction in e′ and increase in E/e′, suggests that the initial cardiac response to treatment may involve changes in myocardial relaxation and ventricular filling before measurable impairment of global systolic performance. This sequence is clinically relevant because it may provide an opportunity for earlier identification of patients at increased risk of subsequent cardiac dysfunction.

Taken together, our results support the use of a multimodal approach for the early detection of cardiac effects during trastuzumab-based therapy. Conventional systolic parameters remained stable, whereas tissue Doppler indices, E/e′, LAVi, and circulating biomarkers demonstrated evidence of early treatment-related changes. The regression analyses further showed that these variables were not merely associated with treatment exposure but contributed independently to the variability in post-treatment filling pressure. The particularly strong associations observed for septal e′ in the echocardiographic model and Gal-3 in the biomarker model suggest that myocardial relaxation and pathways related to myocardial remodeling may represent important components of the early cardiac response to anti-HER2 therapy.

From a clinical perspective, these findings suggest that preserved LVEF and GLS should not necessarily be interpreted as evidence of complete absence of cardiac effects during the early stages of trastuzumab-based therapy. The detection of a significant reduction in tissue Doppler-derived e′ together with an increase in E/e′ may provide additional information regarding early ventricular functional changes. The incorporation of biomarkers such as Gal-3, GDF-15, and hs-cTnI may further improve characterization of individual treatment-related responses. However, these findings should be considered hypothesis-generating and require validation in larger prospective cohorts before they can be translated into specific surveillance or therapeutic recommendations.

Overall, the present study suggests that early diastolic abnormalities may precede detectable systolic dysfunction during trastuzumab-based therapy. The combination of reduced myocardial relaxation velocities, increased estimated filling pressure, and biomarker-associated changes indicates that early cardiac involvement may be better characterized through an integrated echocardiographic and biochemical approach. The regression models, particularly the combined model incorporating baseline E/e′ and treatment-related biomarker changes, provide further support for this multimodal strategy and suggest that it may help identify patients with an early adverse cardiac response while conventional systolic parameters remain preserved.

## 5. Conclusions

In conclusion, the present study suggests that early alterations in left ventricular diastolic function may occur shortly after the initiation of trastuzumab-based therapy, even in the absence of detectable impairment in conventional systolic function or GLS. The reduction in myocardial relaxation velocities together with the increase in estimated left ventricular filling pressure supports the presence of an early treatment-related change in ventricular function.

Echocardiographic and biochemical parameters provided complementary information regarding these early alterations. In particular, treatment-related variations in Galectin-3, GDF-15, and hs-cTnI, together with baseline echocardiographic characteristics, were independently associated with post-treatment E/e′. These findings suggest that integrating assessment of diastolic function with circulating biomarkers may improve the characterization of individual cardiac responses during the early stages of anti-HER2 therapy.

From a clinical perspective, preserved LVEF and GLS should not necessarily be interpreted as evidence of complete absence of early cardiac effects. A multimodal approach combining echocardiographic assessment of diastolic function with biomarkers of myocardial injury and remodeling may help identify patients with early treatment-related cardiac changes. However, given the observational design and limited sample size, these findings should be considered hypothesis-generating and require validation in larger prospective multicenter studies before informing specific surveillance strategies or therapeutic interventions.

## Figures and Tables

**Table 1:** Baseline clinical, demographic, and echocardiographic characteristics according to trastuzumab-based therapy exposure.

	HER2-Positive Group (48 *p*)	HER2-Negative Group (44 *p*)	
Age (years)	64.13 ± 12.72 (61.49–66.76)	65.17 ± 8.50 (62.23–67.45)	0.64
SBP	141.03 ± 17.14	142.00 ± 19.17	0.83
DBP	87.55 ± 17.61	88.96 ± 11.70	0.41
HR	66.37 ± 19.65	64.54 ± 27.48	0.73
LA	34.00 (30.92–35.77)	33.55 (31.01–34.09)	0.76
LA area	13.74 (11.3–17.2)	13.95 (11.9–18.2)	0.15
IVS	10.40 (9.85–10.95)	10.23 (9.53–10.92)	0.56
LVPW	9.97 (9.75–10.95)	9.85 (9.37–10.32)	0.65
LVSDs	28.00 (27.54–30.90)	28.00 (17.00–30.35)	0.22
LVDD	41.00 (39.34–45.32)	41.50 (40.00–45.00)	0.36
RV	30.00 (27.34–33.61)	30.00 (29.34–32.66)	0.74
LVEF	62.8 (55.57–64.80)	63.20 (57.00–63.32)	0.42
MAPSE	15.00 (15.00–16.83)	14.75 (13.43–16)	0.36
TAPSE	22.00 (19.38–23.61)	21.00 (20.00–22.61)	0.86
IVRT	97.38 (74.2–99.03)	98.67 (72.9–99.87)	0.25
GLS	19.35 (18.21–20.45)	19.45 (18.42–20.74)	0.75
DT	210.00 (198.16–233.96)	209.50 (205.35–243.96)	0.13
E wave	82.30 (78.67–89.53)	79.00 (77.23–86.08)	0.24
A wave	76.30 (72.72–81.36)	75.25 (74.45–85.18)	0.99
E/e’	7.46 (6.01–7.97)	7.58 (5.81–9.92)	0.18

**Table 2:** Early biomarker and echocardiographic changes after the first trastuzumab plus docetaxel cycle.

Parameters	Before First Cycle	After First Cycle	Significance
NTproBNP (pg/mL)	102.50 (89.40–123.6)	121.75 (105.00–124.30)	0.064
cTn (hsTn) (ng/L)	24.60 (13.60–34.4)	25.71 (18.24–26.2)	0.06
GDF-15 (pg/mL)	49.20 (36.72–316.16)	49.70 (46.72–416.20)	0.054
Gal 3 (ng/mL)	10.96 (7.87–15.21)	13.05 (12.63–16.30)	0.058
LVEF (%)	62.8 (55.57–61.00)	61.7 (53.2–59.8)	0.46
GLS (%)	19.35 (18.21–20.45)	18.93 (18.02–19.67)	0.53
E/e’ media	7.46 (6.01–7.97)	8.50 (8.11–15.16)	<0.001

**Table 3:** Comparison of diastolic echocardiographic parameters before and after the first trastuzumab plus docetaxel cycle in patients with and without diastolic dysfunction.

	Patients Without Treatment-Emergent Diastolic Abnormalities			Patients with Treatment-Emergent Diastolic Abnormalities		
	Baseline	After First Cycle	*p*	Baseline	After First Cycle	*p*
E wave	82.30 (78.67–89.53)	81.2 (79.3–88.62)	0.067	82.42 (78.45–88.43)	80.36 (77.12–86.83)	0.045
A wave	76.30 (72.72–81.36)	77.23 (73.12–82.41)	0.089	77.14 (72.41–82.34)	79.23 (73.17–83.67)	0.048
E/A	1.07 (1.03–1.1)	0.92 (0.81–0.98)	0.055	1.02 (0.98–1.1)	0.9 (0.73–0.96)	0.032
E/e’media	6.20 (6.03–7.36)	6.78 (6.16–7.73)	0.007	11.5 (9.9–12.16)	15.56 (15.3–16.04)	<0.0001
LAVi (mL/BSA)	22.34 (20.33–27.85)	22.86 (20.21–28.34)	0.89	22.55 (20.13–29.11)	25.63 (22.34–35.1)	0.013

**Table 4:** Distribution of estimated left ventricular filling pressure categories before and after the first trastuzumab plus docetaxel cycle.

	HER2plus (48 Patients)		*p*
E/e’	Baseline	After treatment	
<8	83.3% (40 patients)	16.6% (8 patients)	*p* < 0.0001
8–15	16.6% (8 patients)	50.0% (24 patients)	*p* < 0.0001
≥15	0.0%	33.3% (16 patients)	*p* = 0.0170

**Table 5:** Multiple linear regression models for predicting post-treatment E/e’ ratio using echocardiographic and biochemical parameters for 48 patients receiving trastuzumab-based therapy.

**Model/Predictor**	**B**	**SE**	**95% CI for B**		**R Partial**	***p***
**Model 1. Echocardiographic model**						
Constant	19.1688	1.0455	17.062; 21.276		—	<0.0001
DT	0.0222	0.0039	0.015; 0.030		0.657	<0.0001
RV-RA pressure gradient	0.0684	0.0178	0.032; 0.104		0.501	0.0004
Septal e’	−0.7191	0.0779	−0.876; −0.562		−0.812	<0.0001
**Model 2. Log-transformed echocardiographic model**						
Constant	1.5217	0.05252	1.416; 1.628		—	<0.0001
DT	0.00144	0.000194	0.00106–0.00183		0.749	<0.0001
RV-RA pressure gradient	0.00267	0.00089	0.00087; 0.00447		0.411	0.0046
Septal e’	−0.0358	0.004	−0.044; −0.028		−0.810	<0.0001
**Model 3. Biomarker model**						
Constant	8.5072	0.234	8.036; 8.978		—	<0.0001
hs-cTnI (hsT21)	0.0196	0.00437	0.0108; 0.0284		0.560	0.0001
Gal-3 (Gal21)	0.00096	0.000105	0.00075; 0.00117		0.810	<0.0001
GDF-15 (GDF21)	0.000697	0.00028	0.00014; 0.00126		0.355	0.0154
**Model 4. Combined model**						
Constant	6.7213	0.5289	5.655; 7.787		—	<0.0001
hs-cTnI (hsT21)	0.01606	0.00398	0.0080; 0.0241		0.525	0.0002
Gal-3 (Gal21)	0.00073	0.000113	0.00049; 0.00094		0.698	<0.0001
GDF-15 (GDF21)	0.00066	0.000245	0.00016 to 0.00115		0.379	0.0102
Baseline E/e′	0.2003	0.05460	0.09014 to 0.3104		0.488	0.0007
**Model**	**R^2^**	**Adjusted R^2^**	**Multiple R**	**Residual SD**	**F**	***p***
Echocardiographic	0.765	0.749	0.875	1.272	47.81	<0.001
Log-transformed echocardiographic	0.789	0.774	0.888	0.064	54.70	<0.001
Biomarker	0.725	0.706	0.851	1.378	38.58	<0.001
Combined	0.790	0.771	0.889	1.217	40.48	<0.001

Abbreviations: B, unstandardized regression coefficient; CI, confidence interval; SE, standard error; R^2^, coefficient of determination; R partial—partial correlation coefficient; SD, standard deviation; hs-cTnI, high-sensitivity cardiac troponin I; Gal-3, galectin-3; GDF-15, growth differentiation factor 15; E/e′, ratio of early transmitral filling velocity to early diastolic mitral annular velocity. All models were fitted in 48 patients receiving trastuzumab-based therapy (HER2plus = 1). Model 2 used the natural logarithm of post-treatment E/e′ as the dependent variable.

## Data Availability

The datasets presented in this article are not readily available due to privacy restrictions.
